# Online COVID-19 Misinformation and Infodemic Governance During the Peak Pandemic Period: Scoping Review of Social Media Research From 2020 to 2022

**DOI:** 10.2196/79434

**Published:** 2026-10-29

**Authors:** Yang Liu, Zhiying Yue

**Affiliations:** 1 Department of Computer Information Systems, School of Business, North Carolina Central University, 1801 Fayetteville St, Durham, NC, 27707, United States, 1 9195305276; 2 Digital Wellness Lab, Boston Children’s Hospital, Harvard Medical School, Boston, MA, United States

**Keywords:** COVID-19, misinformation, infodemiology, social media, infodemic management, public health communication, natural language processing, machine learning, scoping review

## Abstract

**Background:**

During the COVID-19 pandemic, social media platforms became major channels for public health communication and public sensemaking, but they also enabled the rapid circulation of rumors, false claims, conspiracy narratives, and misleading health information. Research on online COVID-19 misinformation expanded rapidly during the peak pandemic period, yet the evidence base was fragmented across public health, computing, communication, and information science.

**Objective:**

This scoping review synthesizes empirical research on online COVID-19 misinformation on social media during the peak pandemic period from February 2020 to October 2022. It examines how misinformation was conceptualized, which platforms and public health contexts were emphasized, and how computational methods shaped the evidence base for infodemic management. Its distinct contribution is an integrated comparison of conceptual definitions, thematic and platform concentration, the computational research pipeline, and the gap between detection and actionable infodemic governance.

**Methods:**

We conducted a PRISMA (Preferred Reporting Items for Systematic Reviews and Meta-Analyses)-informed scoping review of empirical studies indexed in PubMed and IEEE Xplore in November 2022 using COVID-19, social media platforms, and misinformation-related terms. Eligible reports were English-language, peer-reviewed empirical studies published between February 2020 and October 2022 that analyzed social media data using computational, quantitative, or social media analytic methods; the language restriction applied to the publication, not to the underlying dataset. The final synthesis included 100 studies selected from 1688 records.

**Results:**

Within the included corpus, the literature was concentrated around vaccine-related discourse, public health and social measures-related misinformation, and text-based data from X (X Corp), formerly known as Twitter. Studies commonly used misinformation detection, classification, sentiment analysis, topic modeling, and network analysis. Fewer studies examined diffusion pathways, source tracing, cross-platform movement, intervention effectiveness, or platform governance. The evidence base remains more developed for detecting and describing misinformation than for evaluating actionable responses to it.

**Conclusions:**

Peak-pandemic COVID-19 misinformation research produced important computational and empirical knowledge, but it remains detection-centered and unevenly connected to public health action. Future infodemiology research should link computational outputs to intervention design, risk communication, fact-checking workflows, platform moderation, multilingual and multimodal analysis, and the evaluation of infodemic governance strategies.

## Introduction

Social media platforms have become central infrastructures for digital information production, circulation, and consumption. Their participatory affordances, algorithmic curation mechanisms, and networked diffusion structures enable rapid information exchange across large and diverse publics [1-3]. At the same time, these same features can accelerate the spread of misinformation, amplify unverified claims, and make misleading information appear credible through repetition, engagement, and social endorsement [4-6]. Online misinformation is not only a problem of false content but also a broader digital information problem shaped by the interaction of users, platforms, algorithms, institutions, and communication environments [7,8].

The COVID-19 pandemic provides a critical empirical setting for examining this problem. During the pandemic, social media platforms became essential channels for public health communication, institutional messaging, community discussion, and real-time public response [9-12]. However, they also facilitated the rapid circulation of rumors, conspiracy theories, misleading claims, and false information concerning the virus, its origins, prevention measures, treatments, vaccines, and public health policies [13,14]. Because COVID-19 combined high uncertainty, high information demand, rapidly changing scientific evidence, and widespread platform-mediated communication, it offers a particularly important context for understanding how online misinformation is produced, circulated, measured, and governed.

Researchers have responded to this challenge by using social media data and computational methods to study COVID-19 misinformation at scale [15]. Natural language processing (NLP), machine learning (ML), deep learning (DL), topic modeling, sentiment analysis, and network analysis have been used to detect misinformation, classify content, identify themes, examine affective responses, and monitor public discourse [16-20]. These methods have generated valuable knowledge about online misinformation during a major public health crisis. However, the literature remains difficult to synthesize because studies differ substantially in how they define misinformation, which platforms and populations they examine, what forms of social media data they collect, and which computational techniques they apply.

This gap motivates the present peak-pandemic scoping review. This peak-pandemic scoping review integrates technical and public health research by examining COVID-19 misinformation as an infodemiology and online health information domain during the intensive period from February 2020 to October 2022. The review does not claim to cover the entire postpandemic misinformation literature; instead, it focuses on the period in which COVID-19 misinformation research rapidly developed around urgent public health uncertainty, high-volume social media data, and computational monitoring. Specifically, we review 100 empirical studies selected from 1688 records identified through the targeted database searches. We analyze how this literature conceptualizes misinformation, what social media contexts it emphasizes, and how computational methods shape what researchers are able to observe, measure, and act upon. In this review, infodemic governance refers to the sociotechnical arrangements through which platforms, institutions, technologies, and users shape the visibility, credibility, correction, moderation, and public health use of online information. This definition allows us to examine misinformation not only as content to be detected but also as an information problem to be managed across platform, institutional, technological, and public communication settings. The distinctive contribution is the joint analysis of conceptual definitions, thematic and platform coverage, the end-to-end computational research pipeline, and the distance between analytic outputs and governance action within one focused corpus. The review is guided by 3 research questions (RQs):

- RQ1: How was COVID-19 misinformation on social media conceptualized and operationalized in empirical infodemiology and online health information research during the peak pandemic period?
- RQ2: What themes, platforms, populations, and online public health information contexts were emphasized or underexamined in this literature?
- RQ3: How were NLP, ML, and related computational methods used to study online misinformation, and what limitations did these methods create for intervention and infodemic governance?

By addressing these questions, this study makes 3 main contributions:

- It clarifies the peak-pandemic conceptual landscape of online COVID-19 misinformation research by synthesizing how misinformation, disinformation, fake news, rumors, and infodemic-related content were defined and operationalized.
- It maps the empirical structure of the literature by identifying dominant themes, platform concentrations, population coverage, and underexamined online contexts during the 2020‐2022 review window.
- It reframes detection-centered computational misinformation research as part of a broader sociotechnical infodemic governance agenda, showing why detection approaches need to be complemented by research on diffusion, cross-platform movement, source tracing, intervention effectiveness, platform governance, and public health communication.

A central challenge in online misinformation research is conceptual inconsistency. Across the literature, terms such as misinformation, disinformation, fake news, rumor, conspiracy theory, and infodemic are often used in overlapping ways, even though they refer to related but analytically distinct phenomena. The COVID-19 pandemic brought particular attention to the concept of an infodemic, understood as the rapid spread of misinformation, rumors, fake news, and stigma during disease outbreaks [21]. In this study, we defined misinformation as false or misleading information regardless of whether there is intent to deceive [22]. This definition distinguishes misinformation from disinformation, which refers more specifically to false or misleading information deliberately created or shared to deceive, manipulate, or cause harm.

The distinction between misinformation and disinformation is important because intent, harm, and information accuracy have different implications for measurement, labeling, moderation, and intervention. During the COVID-19 crisis, some individuals unintentionally circulated inaccurate claims or anecdotes, whereas others intentionally promoted deceptive narratives. To establish conceptual clarity, we adopt these definitions throughout the study.

This distinction is further supported by prior efforts to classify misleading content more precisely. For example, the information disorder framework of Wardle [7] identifies multiple forms of mis- and disinformation, ranging from satire or parody, which may mislead despite lacking harmful intent, to false context and fully fabricated content designed to deceive. Such typologies clarify that problematic information online is not a single phenomenon. Rather, it spans multiple forms that vary in intent, construction, context, and potential harm. This broader conceptualization is important for understanding how COVID-19 misinformation was studied across social media platforms and how different operational definitions shaped the empirical literature.

When describing individual studies, we retained the source authors’ labels, including fake news, rumor, misinformation, and disinformation, rather than retroactively relabeling their datasets. These labels are not treated as interchangeable. Misinformation serves as the umbrella term for synthesis, whereas disinformation is reserved for cases with evidence of deceptive intent. Opinions, personal experiences, fears, and hesitancy are not themselves misinformation unless they contain a verifiably false or misleading factual claim.

Social media platforms are central infrastructures for information dissemination. Their role is inherently double-edged: they enable rapid access to diverse information, but they also facilitate the circulation and amplification of unverified rumors and misleading narratives [23]. This challenge is intensified by algorithmic curation, which prioritizes content partly based on user preferences and prior behavior [4]. As a result, users may be disproportionately exposed to information consistent with their existing views, while conflicting evidence receives less visibility. Prior research has linked this dynamic to echo chambers and greater polarization [5,6]. In such environments, misinformation can spread more easily, gain legitimacy through repetition, and become more resistant to correction.

The public health consequences of this dynamic are significant. Misinformation can undermine coordinated societal responses during health crises, thereby worsening epidemic conditions [8]. In a review of health-related misinformation published between 2012 and 2018, Wang et al [23] found that vaccination was the most frequently studied topic. Their review further suggested that social media may intensify vaccine hesitancy by granting misinformation disproportionate visibility and perceived credibility, thereby increasing public skepticism and resistance to vaccination. During COVID-19, this broader problem became especially visible. False and misleading claims concerning the virus’s origins, transmission, prevention, treatment, and consequences spread rapidly across platforms. These narratives obscured accurate health guidance, encouraged harmful practices, and aggravated both physical and mental health burdens worldwide [24].

At the same time, the scale and structure of social media data create both opportunity and difficulty for researchers. These datasets are large, heterogeneous, and often multimodal, comprising text, images, and video. While such characteristics make social media valuable for studying public discourse during crises, they also increase analytic complexity and measurement risk. Noise, ambiguity, and misinformation make it difficult to extract valid signals for public health and platform decision-making. In response, researchers have increasingly relied on computational social science approaches [15]. NLP methods, such as sentiment analysis, named-entity recognition, and topic modeling, are used to characterize discourse and identify misinformation-related narratives at scale. ML and DL approaches support detection and classification tasks, while network analysis helps examine diffusion patterns and influence structures. These approaches offer analytical and operational value, including support for warning systems and content moderation. However, their usefulness depends not only on technical performance but also on careful evaluation of their broader sociotechnical implications.

Taken together, these conditions justify a structured and auditable synthesis of research at the intersection of social media, misinformation, and COVID-19 during the peak pandemic period from 2020 to 2022. Such a review can clarify what has been studied, identify which computational approaches have dominated the literature, and reveal where important evidence gaps remain. By doing so, it can provide a stronger foundation for the design, evaluation, and governance of human-centered misinformation management during public health emergencies.

## Methods

### Study Design and Reporting Framework

This study used a scoping review approach to synthesize a focused corpus of empirical social media studies at the intersection of COVID-19, misinformation, and computational analysis. The objective was to identify conceptual, empirical, and methodological patterns. The review does not estimate aggregate effect sizes. We reported the available search criteria, eligibility rules, and aggregate counts. PRISMA (Preferred Reporting Items for Systematic Reviews and Meta-Analyses) 2020 and PRISMA-ScR (Preferred Reporting Items for Systematic Reviews and Meta-Analyses extension for Scoping Reviews) informed the reporting of the search and selection process, to the extent applicable to this non–meta-analytic scoping review (Checklist 1). The available documentation includes database-level identification totals, database-specific eligibility totals before cross-database deduplication, the cross-database duplicate count, and the final included corpus.

### Search Strategy Development

Our exploration of the landscape of social media “misinformation” began with an initial exploratory search in November 2022, using digital repositories such as Google Scholar and our digital library databases. Using “misinformation” and “COVID-19” as our starting keywords, we used a dynamic method to adjust our search query. We initially used various query approaches with these terms to capture the relevant literature (n=20). As we progressed, we identified additional keywords that frequently co-occurred in our preliminary findings. These new keywords were then incorporated into our primary search. This evolving approach of keyword discovery and inclusion not only broadened our literature base but also sharpened our research focus, aiding in the precise formulation of our RQs, refinement of terminology, and establishment of specific inclusion and exclusion criteria for our study.

### Information Sources and Search Strategy

Building upon the exploratory phase, we conducted a targeted search in PubMed and IEEE Xplore. These 2 databases were selected because they jointly cover 2 major streams of COVID-19 misinformation research relevant to this review. PubMed captures biomedical, public health, and health communication studies that examine misinformation as a public health and infodemic problem [25], whereas IEEE Xplore captures computing, engineering, AI, NLP, and ML studies that examine misinformation through computational detection, classification, and analysis. By combining these 2 repositories, our goal was to construct a focused corpus of empirical studies that reflects the intersection of public health misinformation and computational social media analysis. We acknowledge that this search strategy does not cover all possible information science, communication, or social science databases; therefore, the review should be interpreted as a structured synthesis of a focused multidisciplinary corpus rather than a comprehensive systematic review of all COVID-19 misinformation research.

### Eligibility Criteria

During the targeted repository search, we used a tripartite context-platform-topic keyword strategy. The sample was restricted to peer-reviewed empirical studies based on original data and analysis; reviews, commentaries, survey papers, and meta-analyses were excluded. Eligible reports were published in English between February 2020 and October 2022 and used computational, quantitative, or social media analytic methods. The English-language criterion applied to the publication, not to the language of the underlying social media data; studies analyzing non-English datasets remained eligible. Studies were included only when the searchable title, abstract, or keyword/index-term fields contained at least one term from each of the three categories below, subject to the field structure available in each database.

1. Context: the study had to concern itself with the COVID-19 pandemic. The COVID-19 context was identified from the searchable title, abstract, or keyword/index-term fields using the following terms: “covid-19,” “covid19,” “covid,” “coronavirus,” and “acute respiratory syndrome.”
2. Platform: we identified the platform as social media by keywords such as “social media,” “social network,” “Twitter,” “tweet,” “Reddit,” “Facebook,” or “Weibo.” Our search also included keywords for “Instagram,” “Snapchat,” and “YouTube.” Because the primary goal of the current study addressed text-centered social media misinformation research, studies focused solely on nontextual image or video content outside this scope were excluded. The final sample nevertheless included studies addressing misinformation on YouTube (Google LLC; n=2) and Instagram (Meta Platforms, Inc; n=2) when they met the review criteria.
3. Topic: the exact misinformation-related search terms were “misinformation,” “disinformation,” “fake news,” “rumor,” and “infodemic.” No additional adjacent labels, such as “inaccurate information,” “unreliable information,” or “low-quality information,” were included in the targeted search. The search identifies studies that self-described their focus using the specified misinformation vocabulary; studies using only adjacent terminology may have been missed. The reported terms describe the recoverable component terms of the targeted search; a complete database-exported record of the original field-specific query syntax was not retained. Additional details on the search logic, database-specific record counts, supplementary title/abstract search audit, and PRISMA-ScR–informed reporting are provided in Multimedia Appendix 1.

The 2 researchers divided the initial screening tasks by database; one researcher screened records retrieved from IEEE Xplore, and the other screened records retrieved from PubMed. Following the initial screening, every inclusion and exclusion decision was verified by the other researcher against the prespecified eligibility criteria. This verification was conducted sequentially and was not independent of the initial screening decision. Disagreements were resolved through discussion and consensus. No disagreement remained unresolved. Therefore, third-party adjudication was not used. No formal interrater reliability statistic was calculated.

### Data Extraction and Synthesis

Each researcher extracted data from the studies retained from their assigned database using the same structured extraction framework. The framework recorded publication year, title, source link, methodological approach, platform, topic, inclusion or exclusion decision, major RQ, specific misinformation topic, sample selection strategy, sample size, statistical analyses, model strengths and weaknesses, and conclusion. Data extraction was divided by database and was not performed independently in duplicate. Accordingly, no formal interrater reliability coefficient, such as Cohen kappa, was calculated.

The thematic synthesis used the common extraction framework as its evidentiary basis. Information recorded under the topic, major RQ, specific misinformation topic, methodological approach, platform, sample selection, statistical analysis, model strengths and weaknesses, and conclusion fields was compared across the included studies and organized around the 3 review questions. The resulting themes were used to identify recurring conceptual definitions, public health topics, platform contexts, data acquisition strategies, computational methods, and governance-relevant gaps. Because the included studies used heterogeneous data sources, analytical methods, and outcome measures, we used a conceptual synthesis and did not estimate aggregate effect sizes. The themes and analytical lenses were not treated as mutually exclusive study categories. The retained records do not document independent duplicate thematic coding or a formal reliability assessment for the thematic synthesis; accordingly, the resulting structure should be interpreted as an author-developed conceptual synthesis rather than a validated study-level multilabel coding scheme. The 2 researchers jointly reviewed the proposed thematic organization and resolved differences in interpretation through discussion and consensus.

## Results

### Study Selection and Sample Characteristics

The process of literature search and identification is shown in Figure 1.

**Figure 1.**
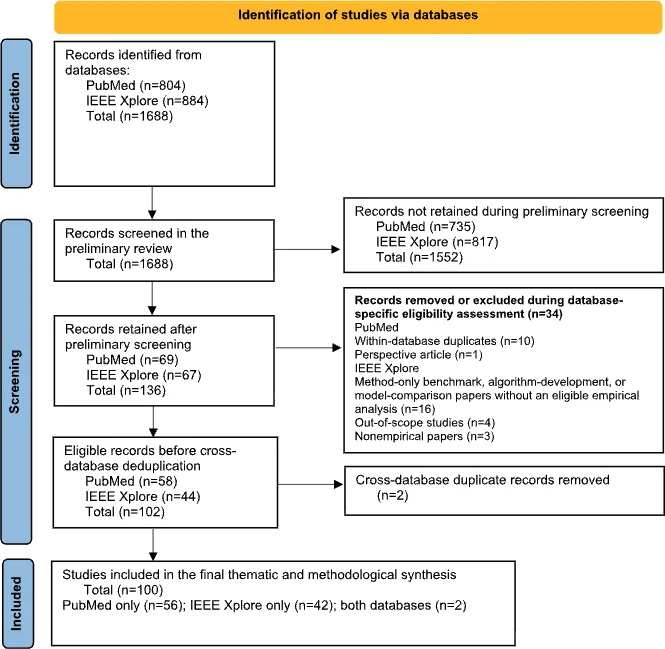
Overview of database identification, aggregate screening and eligibility assessment, cross-database deduplication, and final study selection.

The targeted database searches identified 1688 records, comprising 804 records from PubMed and 884 records from IEEE Xplore. Preliminary screening retained 69 PubMed records and 67 IEEE Xplore records for detailed assessment; 735 PubMed records and 817 IEEE Xplore records were not retained at this stage. Within the PubMed set, 10 within-database duplicate records were removed, leaving 59 unique records. One perspective article was subsequently excluded because it did not report eligible original empirical research, leaving 58 eligible PubMed studies. Among the 67 IEEE Xplore records retained after preliminary screening, 23 were excluded during eligibility assessment: 16 were benchmark, algorithm-development, or model-comparison papers lacking an eligible empirical analysis of COVID-19 misinformation on social media; 4 concerned non-COVID-19 topics, general fake news, or platforms or topics outside the prespecified scope; and 3 were theoretical, policy-oriented, research-agenda, or other nonempirical papers. This process left 44 eligible IEEE Xplore studies. After cross-database deduplication removed 2 duplicate records, the final synthesis comprised 100 unique empirical studies: 56 identified only through PubMed, 42 identified only through IEEE Xplore, and 2 identified through both databases. The retained screening documentation supports the reporting of database-level identification totals, aggregate preliminary-screening counts, within-database duplicate removal, principal eligibility exclusion categories, the cross-database duplicate count, and the final included corpus. However, a complete contemporaneous item-level screening log and record-level exclusion reasons for every record not retained during preliminary screening were not preserved. Accordingly, Figure 1 presents an aggregate summary of study selection and should not be interpreted as a complete PRISMA flow diagram.

The largest number of articles were published in 2021 (n=44), followed by a comparable number in 2022 (n=42), and fewer articles in 2020 (n=14). Most studies analyzed data in X (X Corp), formerly known as Twitter (n=58, 58%), followed by multiplatform/cross-platform (n=25, 25%) and other individual platforms (Facebook; Meta: n=5; Weibo; Sina.com Technology (China) Co, Ltd: n=4; Reddit; Reddit, Inc: n=3; Instagram; Meta: n=2; YouTube; Google: n=2; LINE; LY Corporation: n=1).

### Conceptualization and Typology of Misinformation

#### Overview

To frame our analysis, it is important to clarify how misinformation is conceptualized across the reviewed studies. We found that authors do not always use consistent definitions or typologies of misinformation and disinformation. Through our review, we identified several categories of misinformation-related content that differ in both the nature of the falsehood and the intent underlying it. Drawing on prior frameworks, particularly the information disorder framework proposed by Wardle [7], we developed a working typology that provides a conceptual framework for interpreting the forms of misinformation and disinformation described in the reviewed literature. It was not used to assign all 100 studies to mutually exclusive categories or to generate category-specific frequencies. The typology classifies content and acts of dissemination; it does not classify unverifiable subjective experiences. Sender intent differentiates misguided, opportunistic, and malicious dissemination when intent can be supported; receiver interpretation or behavior is a downstream outcome and does not itself determine whether content is misinformation. Because intent is often difficult to infer from content alone, these categories should be treated as conceptual distinctions rather than definitive judgments about individual users. Figure 2 presents this typology along 2 conceptual dimensions: intent to deceive and potential harm.

**Figure 2.**
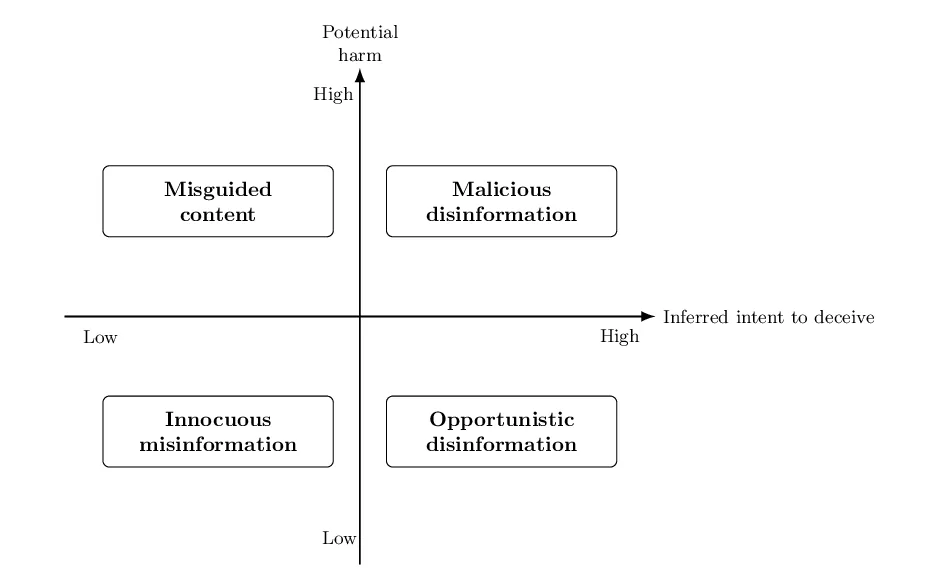
An author-developed conceptual typology of misleading information by inferred sender intent and potential harm.

#### Innocuous Misinformation

This category encompasses factually false or misleading content shared without the intent to deceive or cause harm [7]. It may arise from satire, parody, reporting errors, humor, or sincere misunderstanding. Personal fears, concerns, opinions, and experiences are not misinformation unless they contain a fact-checkable false or misleading claim. This distinction is particularly important when interpreting vaccine-related social media discourse. For example, Stevens et al [26] examined anxiety, anger, sadness, and incivility in tweets about COVID-19 vaccine mandates but did not assess the factual accuracy of those tweets; therefore, the emotional expressions analyzed in that study should not themselves be classified as misinformation. By contrast, a claim such as “ice cream prevents COVID-19 infection,” if repeated as a joke or in a sincere mistake without deceptive intent, would constitute innocuous misinformation.

#### Misguided Content

This category involves inaccurate or distorted information shared by individuals who sincerely believe it to be true. Rumors and unverified claims, especially common during crises when reliable information may be scarce, are central to this category [27]. For example, claims such as “mRNA alters a person’s DNA” or “the COVID-19 vaccine contains tissue from aborted fetuses” fall into this category when they are spread by individuals who mistakenly believe them to be accurate.

#### Opportunistic Disinformation

This category refers to false or misleading information deliberately disseminated to obtain personal, political, professional, or commercial advantage [28-30]. Unlike malicious disinformation, its primary objective is benefit seeking rather than the deliberate infliction of substantial harm, although harmful consequences may still occur [28]. Such content may exploit timely events, public attention, or existing concerns to attract engagement, influence perceptions, generate revenue, or promote interests. Examples include clickbait, exaggerated claims, and commercially motivated false or misleading health or product claims [29,30]. Its placement in the lower-harm quadrant indicates comparatively lower or more indirect potential harm than malicious disinformation; it does not imply that the consequences are necessarily harmless or minor.

#### Malicious Disinformation

This category refers to content that is knowingly and deliberately disseminated to mislead, distort public perceptions, or cause harm. It may include fabricated content, manipulated content, imposter content, and other misleading messages when there is evidence of deliberate deceptive intent [31]. The false claim linking fifth-generation (5G) mobile networks to the spread of COVID-19 is an example of a fabricated COVID-19 misinformation narrative with the potential to mislead the public and provoke fear [32]. It should be classified as malicious disinformation only when the available evidence supports knowing and deliberate dissemination; the falsity or potential harmfulness of the claim alone does not establish sender intent.

### Thematic and Platform Concentration

#### Overview

To address RQ2, we examined the online public health information contexts and thematic emphases represented in the reviewed studies. Instead of treating the categories as mutually exclusive groups of studies, we organize recurring findings into 2 parallel topic domains (general COVID-19 and public health/social measures and vaccination/vaccine uptake) and a set of cross-cutting analytical lenses. The domains and lenses can overlap conceptually; for example, amplification and cultural context may apply to both topic domains. The available review records do not support reliable per-theme study counts or an overlap matrix, so Figure 3 is presented as a conceptual map rather than a quantitative classification. This distinction is important for infodemiology because the observed concentration of topics and platforms shapes what kinds of misinformation become visible, which communities are studied, and what governance implications can be drawn.

**Figure 3.**
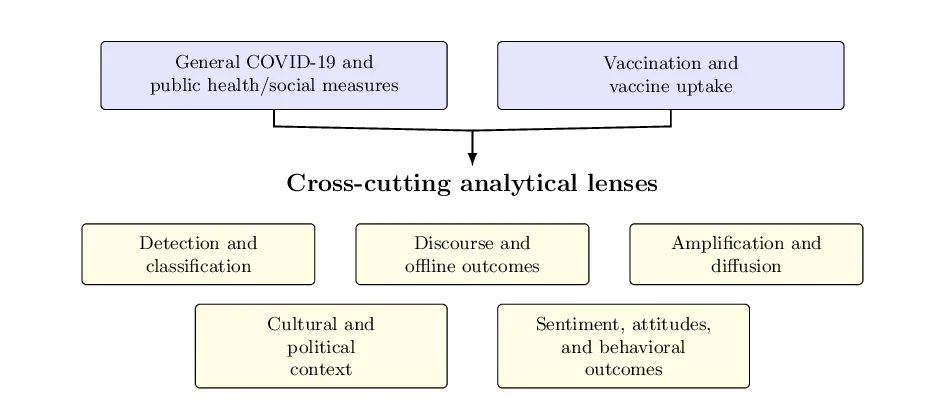
Conceptual map of 2 parallel topic domains and cross-cutting analytical lenses in the reviewed literature; domains and lenses are not mutually exclusive.

#### General COVID-19 and Public Health/Social Measure–Related Misinformation

##### Overview

This topic domain captures studies of general COVID-19 misinformation and misinformation concerning public health and social measures, including nonpharmaceutical measures such as mask-wearing, distancing, isolation, and related prevention guidance. It includes research on how misleading information shapes, reflects, or interacts with public attitudes, discourse, and behavior. Studies in this domain commonly use content analysis, sentiment analysis, ML, and network-based approaches to analyze large-scale social media data.

##### Detection and Classification

This cross-cutting analytical lens focuses on the identification, classification, and interpretation of misinformation at scale. The studies grouped here primarily aim to develop or apply computational approaches that can detect misleading content and characterize its patterns across social media environments. In this sense, their central contribution lies not only in identifying false or misleading content but also in improving the methodological capacity to analyze misinformation systematically. For example, Bangyal et al [33] proposed a high-accuracy approach for sentiment analysis of COVID-19 fake news on Twitter. Their study compared multiple ML and DL techniques, including naive Bayes (NB), AdaBoost, k-nearest neighbors (KNN), convolutional neural networks (CNN), long short-term memory (LSTM), recurrent neural networks (RNN), and gated recurrent units (GRU). By evaluating a range of models on COVID-19 fake news data, the study illustrates how advanced computational techniques can be used to strengthen large-scale misinformation detection. Rastogi and Bansal [31] developed an integrated approach combining social science theory with computational analysis to distinguish among real information, disinformation, and satire. Their framework is notable because it extends beyond binary authenticity classification to consider communicative intent, using features such as frequency, complexity, uncertainty, sentiment, and subjectivity. Studies examined through this analytical lens deepen understanding of misinformation patterns, improve classification strategies, and clarify the analytical dimensions through which misinformation can be examined during global crises.

##### Discourse and Offline Outcomes

This cross-cutting analytical lens concerns relationships between online discourse and offline social or public health outcomes, including whether online signals mirror, anticipate, or coincide with behavior, events, or policy-relevant developments. The core premise is that social media data may function as an indicator of broader public response during a health emergency. For instance, Al-Ramahi et al [34] analyzed a large volume of tweets related to antimask discourse during the COVID-19 pandemic and found that increases in negative mask-related tweets were associated with rises in new COVID-19 cases. Their findings suggest that online sentiment may reflect or coincide with consequential behavioral patterns offline. Jang et al [35] analyzed COVID-19-related tweets and found that identified topics and their temporal trajectories aligned closely with public health promotions and interventions. These studies show that social media can serve as a useful lens through which to observe public concerns, reactions, and behavioral tendencies in near real time. This analytical lens also includes work on rumor-specific discourse that gains traction and produces tangible social consequences. Bahja and Safdar [32] examined the rumor linking 5G networks to the spread of COVID-19. Using social network analysis, they identified key actors involved in spreading the claim and the persuasive strategies used to sustain it. This line of work is important because it demonstrates how specific misinformation narratives can move beyond online circulation and contribute to harmful offline outcomes.

##### Cultural and Political Context

This cross-cutting analytical lens examines how misinformation is shaped by national, cultural, political, and social context. It recognizes that dissemination, uptake, and persistence vary across informational and societal conditions. As a result, comparative analysis becomes essential for understanding the global yet uneven nature of the COVID-19 infodemic. For example, Park et al [36] investigated Twitter discourse related to COVID-19 in 4 Asian countries and found variation in public attention, misinformation patterns, and topic diversity across national contexts. de Oliveira and Albuquerque [37] examined the spread of false information on Twitter in Brazil and highlighted the possible role of cultural context and social isolation in sustaining misinformation within the information ecosystem. These studies suggest that misinformation should not be analyzed as a context-free phenomenon. Instead, its forms and trajectories must be understood in relation to the cultural and social environments in which it is produced, circulated, and interpreted.

### Vaccination and Vaccine Uptake–Related Misinformation

#### Overview

This second topic domain concerns misinformation and related discourse about COVID-19 vaccination, vaccine confidence and hesitancy, and vaccine uptake. It examines not only false or misleading vaccine claims but also the discursive, behavioral, and political environments in which those claims circulate. The following paragraphs emphasize vaccine-related examples while illustrating analytical lenses that can also operate across the general COVID-19 and public health/social measures domain.

#### Detection and Classification in Vaccine-Related Studies

When applied to vaccination-related research, this analytical lens includes studies that identify, classify, and interpret vaccine-related misinformation and closely related forms of public discourse. A central concern in this line of work is to distinguish misinformation from other types of vaccine-related content, such as opinion, news, or general expressions of hesitancy, to better understand the structure of online vaccine discourse. For example, Weinzierl and Harabagiu [38] developed a graph link-prediction approach for the automated detection of COVID-19 vaccine misinformation. Ke et al [39] analyzed more than 646 million COVID-19-related tweets to investigate public health beliefs about mask-wearing and vaccination. Their study emphasized the role of “high-profile cues to action,” such as guidelines and statements from major institutions including the WHO and CDC, in shaping public beliefs and narratives. These studies show that vaccine discourse research is concerned not only with detecting false claims but also with mapping the broader informational and attitudinal landscape within which such claims circulate.

A more specific strand within this analytical lens focuses directly on the detection of vaccine-related misinformation and rumors. Hayawi et al [40] developed ML-based approaches to detect misinformation related to COVID-19 vaccines. Shang et al [41] similarly identified vaccine-related misleading posts on social media using community-contributed COVID-19 fact data (CCFD), while also addressing user privacy concerns. These studies demonstrate the importance of scalable detection methods for identifying misleading vaccine content and supporting timely public health responses.

#### Amplification and Diffusion in Vaccine-Related Studies

A further analytical lens concerns the mechanisms through which vaccine-related misinformation is amplified on social media. These studies extend the analysis beyond the identification of misleading content to examine the relational, discursive, and political processes that enable misinformation to spread, gain attention, and reinforce hesitancy. For example, Guntuku et al [42] examined interaction patterns among users and showed how these dynamics can amplify both misinformation and negative sentiment, thereby intensifying vaccine hesitancy. Sauvayre et al [43] applied ML and NLP techniques to French-language tweets about vaccination and found that political considerations played a significant role in shaping vaccine-related discourse on X, formerly known as Twitter. Their findings suggest that vaccine misinformation does not circulate in a neutral informational environment; rather, it is often embedded in politically charged discussions that influence public interpretation and vulnerability to misleading claims. This line of research is important because it shifts attention from misinformation content alone to the social and political conditions that sustain its visibility and influence.

#### Sentiment, Attitudes, and Behavioral Outcomes in Vaccine-Related Studies

Another analytical lens examines the relationship between online vaccine discourse and real-world vaccination behavior. These studies are especially important because they connect social media analysis to measurable public health outcomes, thereby extending the relevance of online discourse beyond communication patterns alone. Bari et al [44] developed a real-time big data analytics framework using NLP-based sentiment analysis to track vaccine sentiment on Twitter. Their findings showed that negative sentiment increased after vaccine rollout, indicating that public discourse became more polarized over time. At the same time, they found that increases in positive Twitter sentiment were followed by increases in vaccination rates. Cross-correlation analyses across US regions further suggested that Twitter sentiment scores were associated with vaccination rates with an approximate one-week lag. These findings indicate that social media sentiment may function as an early signal of vaccine uptake and highlight the potential value of social media analytics for informing targeted communication and vaccination strategies.

The findings are organized around 2 major topic domains and multiple cross-cutting analytical lenses. This structure is intended to organize the findings; the topic domains and analytical lenses are not mutually exclusive. Cultural context, amplification, and discourse-behavior relationships may apply across both topic domains. Concentration around vaccines and public health/social measures is understandable given the urgency of the pandemic and the significance of vaccine uptake, but it narrows the empirical basis of the corpus. Importantly, the apparent scarcity of multimodal and behavioral evidence partly reflects the eligibility criteria: noncomputational behavioral studies and studies focused solely on nontextual image or video content were excluded. The identified gaps characterize the included corpus, not the entire COVID-19 misinformation literature. Future work should move beyond topic-specific and platform-specific snapshots toward comparative, cross-platform, multilingual, and multimodal analysis.

### Computational Approaches as Online Information Processing Tools

### Data overview

Having examined the thematic and contextual structure of the literature, we now turn to the computational approaches used to study online COVID-19 misinformation. This section addresses RQ3 by synthesizing how researchers collect, prepare, model, and analyze social media data. We interpret these methods not only as technical tools, but also as online information processing mechanisms that shape what researchers can observe, classify, explain, and potentially govern. Across the reviewed studies, the analytical pipeline generally involves data collection, data preparation, and modeling and analysis.

### Data Collection

#### Overview

Data collection is a foundational stage in COVID-19 misinformation research because the choice of platform and acquisition strategy shapes both the scope of analysis and the kinds of claims that can be made. In the reviewed literature, most studies collected their own datasets from social media platforms, while a smaller number relied on publicly available datasets. Across these studies, data collection is structured by 2 core considerations: the selection of platform sources and the criteria used to retrieve relevant data. Together, these decisions determine what forms of discourse enter the dataset and what dimensions of misinformation can subsequently be observed. Figure 4 distinguishes data sources and communication modalities represented in the included studies from modalities that were absent or underrepresented in the corpus. The latter are shown to identify future research opportunities and should not be interpreted as evidence that corresponding studies were included in the reviewed sample.

**Figure 4.**
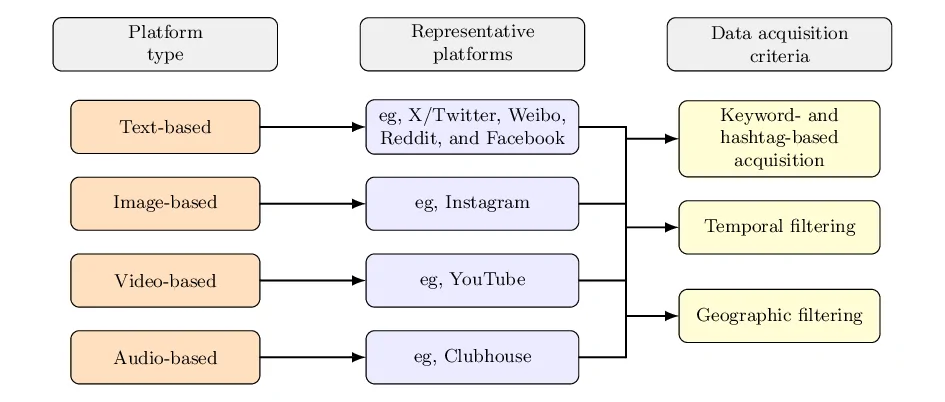
Social media data sources and communication modalities represented in the reviewed literature, together with underrepresented modalities identified as priorities for future research.

#### Data Sources: Social Media Platforms

The reviewed studies drew primarily on major text-based social media platforms. X, formerly known as Twitter, is the most frequently used source [45-49], followed by Weibo [50-53], Reddit [54-56], and Facebook [57-60]. These platforms are particularly prominent because they provide large volumes of user-generated textual data that are well-suited to misinformation analysis, discourse modeling, and computational classification.

X, formerly known as Twitter, is the most extensively used platform in the reviewed literature, largely because it offers high-volume, real-time, and relatively accessible discourse for analyzing rumor propagation, coordinated amplification, sentiment, and credibility. For example, Kirn and Hinders [61] analyzed more than 60 million tweets and used ridge count thresholding to identify coordinated tweetstorms and the communities most responsible for amplifying narratives across the platform. Abdelminaam et al [62] applied ML, DL, and NLP techniques to detect COVID-19 fake news across 4 benchmark datasets. Abe et al [63] used Twitter data to study rumors about toilet paper shortages during the pandemic. Similarly, Al-Rakhami and Al-Amri [64] developed a credibility assessment framework for COVID-19 tweets using 6 ML algorithms. These studies show that X/Twitter is especially useful for examining both large-scale diffusion dynamics and automated misinformation detection.

The dominance of X/Twitter also reflects differential platform data availability. During much of the review window, X/Twitter functioned as an easy-data environment because researchers could obtain large quantities of structured public data through its APIs, whereas platforms such as Facebook offered fewer public data and imposed stricter API limits [65]. Therefore, the number of X/Twitter studies should not be interpreted as a direct measure of that platform’s public health importance or misinformation prevalence.

Weibo is a major source of data for studies focusing on Chinese-language social media environments. Its importance lies in its ability to capture platform-specific discourse within the Chinese context, where rumor dynamics, language use, and interaction patterns may differ from those found on Western platforms. Lu et al [52] constructed a Weibo COVID-19 rumor dataset and proposed a few-shot learning-based fusion model integrating Bidirectional Encoder Representations from Transformers (BERT) embeddings, bidirectional gated recurrent units (Bi-GRUs), and meta-learning. Shi et al [53] used text, user, interaction, and emotion features to build an extreme gradient boosting (XGBoost) rumor detection model that achieved 91% accuracy. These studies suggest that Weibo is both an important regional data source and a useful setting for methodological experimentation in rumor detection.

Reddit occupies a distinct position because its subreddit structure allows researchers to study misinformation within topic-specific communities. Unlike more open broadcasting platforms, Reddit organizes discourse into semi-bounded spaces, making it well-suited to the analysis of community-level narratives, ideological reinforcement, and conspiracy-oriented discussion. Shahsavari et al [66] used Reddit and 4chan posts to identify emerging COVID-19 conspiracy theories through an ML-based graphical narrative discovery pipeline. Bhattacharyya [67] examined r/liberal and r/conservative to analyze the propagation and acceptance of COVID-related misinformation over time. Together, these studies show that Reddit is particularly valuable for understanding how misinformation becomes embedded in community dynamics and polarized discourse.

Facebook is less frequently represented than X/Twitter in the reviewed literature, but it remains an important platform because of its scale and influence on public communication. Research using Facebook data is particularly useful for examining interactions between misinformation, fact-checking, and user engagement. For instance, Xue et al [68] collected more than 12,000 fact-checking Facebook posts related to COVID-19 vaccines and over 122,000 associated comments. Using NLP and statistical analysis, they examined public attitudes, emotional patterns, and linguistic features in the vaccine fact-checking ecosystem. Their findings highlight the importance of third-party fact-checkers and suggest declining uncertainty about vaccines over time.

A smaller subset of studies also incorporates multimedia-sharing environments such as Instagram, LINE, and YouTube. These platforms expand the analytical scope of COVID-19 misinformation research by capturing visual, video-based, or message-chain communication that may not be fully represented in text-centered platforms. Zarei et al [69] published a large Instagram dataset and examined engagement patterns, hashtag use, and user behavior. Wang et al [70] studied rumor groups on LINE using a classification-based clustering approach, while Ginossar et al [71] analyzed YouTube videos shared in vaccine-related tweets and found a strong presence of conspiracy-related content. Although the reviewed literature remains predominantly focused on text-based data, these studies indicate that misinformation research is beginning to extend across multiple communication modalities.

#### Data Acquisition Criteria

Across the reviewed studies, social media data are typically acquired using 3 main criteria: keyword- or hashtag-based queries [72-75], temporal boundaries [76-79], and geographic filters [42,80-82]. These criteria are often used in combination to define the scope of a dataset, align collection procedures with a specific RQ, and facilitate the analysis of large-scale social media data. More importantly, they shape not only what data are collected but also what forms of misinformation become visible to analysis.

Keyword- and hashtag-based acquisition is common in studies focused on topical discourse, rumor detection, and vaccine-related conversation. Hagen et al [77] collected more than 1.3 million tweets using vaccine-related keywords and used network analysis and unsupervised ML to show that vaccine conversations on Twitter were highly polarized. Sharma et al [79] collected 40,000 tweets using COVID-related hashtags and then applied BERT and topic modeling techniques to identify false-information topics. These studies illustrate how keyword selection defines the topical boundaries of the dataset and directly affects the narratives that can be analyzed.

Temporal filtering is important in studies concerned with change over time. Benis et al [76] collected more than 2.7 million tweets across a broad time period to examine how influenza- and COVID-19-vaccination discussions evolved. By delimiting a specific temporal window, such studies can trace changes in attention, sentiment, and misinformation narratives across different stages of the pandemic.

Geographic filtering is especially useful when the goal is to analyze localized misinformation ecosystems or region-specific public discourse. Clamor et al [80] collected tweets from Metro Manila and nearby areas to examine COVID-19 misinformation in the Philippines. Using topic modeling and classification techniques, they identified multiple forms of user activity, including joking, awareness raising, criticism of government responses, and the sharing of preventive advice. This study illustrates how geographic constraints can help isolate context-specific discourse and reveal how misinformation is embedded in local communicative practices.

Overall, data acquisition criteria are not neutral procedural choices. They define the scope of the dataset, shape the patterns that become visible to analysis, and constrain the conclusions that can be drawn. Keyword selection determines topical inclusion, temporal boundaries capture discursive change, and geographic filters enable contextual specificity. These criteria form a key part of the methodological and interpretive boundaries of COVID-19 misinformation research.

### Data Preparation

Because raw social media data are noisy, heterogeneous, and platform-dependent, data preparation is essential for ensuring that subsequent analysis is both reliable and interpretable. User-generated content frequently contains spelling errors, grammatical inconsistencies, abbreviations, vernacular expressions, and platform-specific conventions, all of which complicate systematic analysis. Figure 5 summarizes the data preparation pipeline identified in the reviewed literature. This pipeline generally involves 2 closely related stages: data cleaning and feature engineering. Although these stages are methodologically distinct, they are tightly interconnected. Data cleaning reduces noise, feature engineering shapes content representation, and subsequent modeling depends on the quality of both.

**Figure 5.**
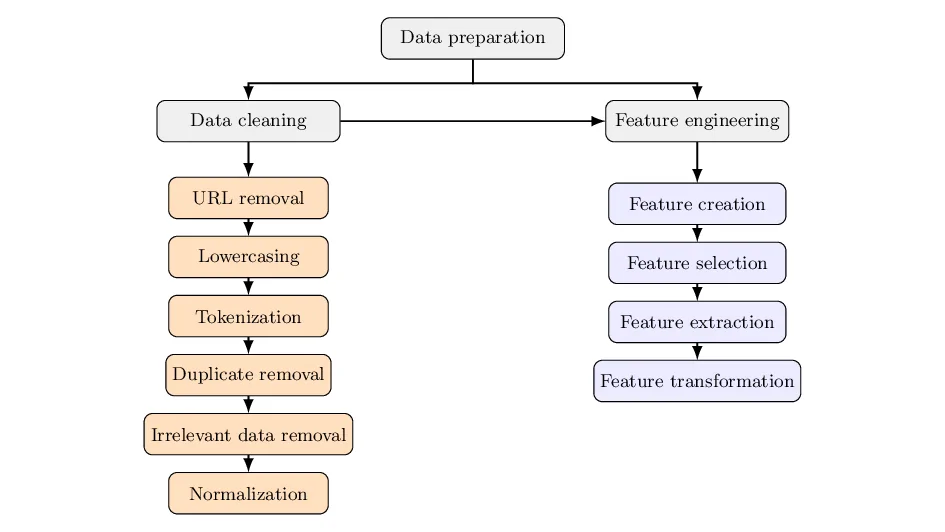
Key stages of data preparation in the reviewed studies, including data cleaning and feature engineering.

Data cleaning is a foundational step in improving dataset quality and analytical usability and is widely used across the reviewed studies [83]. Common techniques include URL removal, lowercasing, tokenization, duplicate removal, elimination of irrelevant elements such as punctuation, stop words, and digits, as well as normalization procedures such as stemming and lemmatization. These steps are intended to reduce noise, standardize textual inputs, and improve downstream model performance. For example, Sharma et al [84] used a COVID-19 dataset collected from Twitter, Facebook, Reddit, and other platforms to classify real and fake news. Their preprocessing pipeline included removing stop words and special characters, applying lemmatization and stemming, tokenizing text, and converting words into vectors. After these steps, they compared several ML models, with bidirectional LSTM (Bi-LSTM) achieving the highest accuracy. Other studies apply cleaning procedures in more targeted ways depending on the linguistic or structural characteristics of the dataset. Shushkevich and Cardiff [85] removed terms that appeared either too rarely or too frequently, thereby reducing sparsity and overrepresentation. Alsudias and Rayson [86], who worked with Arabic tweets, included the removal of non-Arabic words as part of their cleaning process, while Bahja and Safdar [32] replaced abbreviations with full expressions during preprocessing. These studies show that data cleaning is not a uniform procedure but a context-sensitive stage shaped by language, platform, and research objective.

Feature engineering refers to the process of generating, selecting, extracting, and transforming raw data into representations suitable for ML models [87]. In the reviewed literature, this stage plays a central role in determining what aspects of misinformation become analytically visible. Because misinformation is multidimensional, researchers often construct features that capture not only lexical content but also contextual, structural, behavioral, and psycho-linguistic characteristics of posts and users. Some studies emphasize contextualized semantic representations. Chowdhury et al [88] used fine-tuned BERT and RoBERTa models to extract embeddings from COVID-19-related tweets, combined them through stacked embeddings, and passed them through a BiLSTM module before classification. Other studies extend feature engineering beyond message text. Mourad et al [89] analyzed not only tweet content but also user profiles, metadata, and tweet context, showing how the pandemic was exploited to redirect attention to irrelevant topics and false medical information. Michel et al [90] operationalized tweet utility by classifying messages into communicative categories such as caution and advice, doubt and criticism, and rumors and counter-rumors, thereby linking message type to diffusion dynamics.

A further strand of the literature examines the performance implications of different feature extraction and selection techniques [91]. The study compared principal component analysis (PCA) and chi-square feature selection with bag of words (BoW) and term frequency-inverse document frequency (TF-IDF) representations across multiple ML and DL models, finding that TF-IDF and BoW performed better than the selected features and that full preprocessing yielded the strongest results. Mahbub et al [92] proposed a hybrid feature space combining content-based and psycho-linguistic features to analyze rumor propagation on Twitter, showing that psycho-linguistic attributes improved rumor detection accuracy. These studies indicate that feature engineering is not merely a technical intermediary step. It is a substantive analytical stage in which researchers determine how misinformation will be represented, measured, and ultimately interpreted.

### Modeling and Analysis

#### Modeling Overview

Once data have been collected and transformed into analyzable forms, researchers use NLP and ML methods to address a range of analytical objectives. Across the reviewed literature, these objectives cluster around 4 broad areas: topic modeling and thematic analysis [93,94], sentiment and emotion analysis [95-97], false information detection and classification, including misinformation, disinformation, and fake news detection [98-102], and multilingual or cross-lingual analysis [103-106]. These approaches are not mutually exclusive; rather, they often overlap within the same study. Together, they show that COVID-19 misinformation research spans exploratory, interpretive, predictive, and comparative tasks. Figure 6 presents a high-level taxonomy of the major modeling and analysis approaches identified in the reviewed studies.

**Figure 6.**
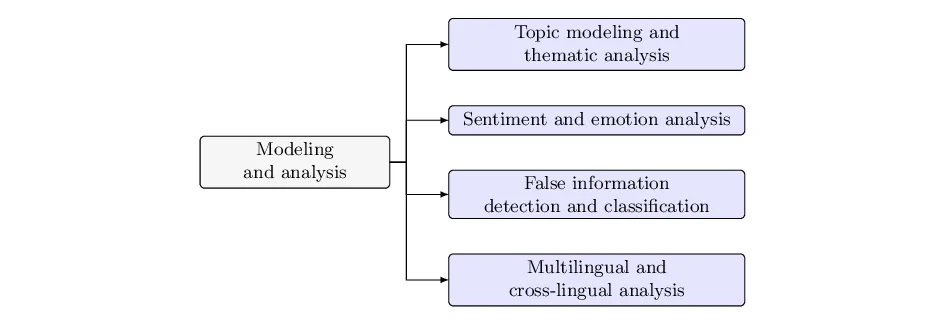
A high-level taxonomy of the major modeling and analysis approaches identified in the reviewed studies.

#### Topic Modeling and Thematic Analysis

Topic modeling and thematic analysis are commonly used to identify recurring narratives, discursive patterns, and user communities associated with COVID-19 misinformation. These approaches are especially useful when the goal is to uncover the substantive structure of discourse rather than merely classify individual posts. Kalantari et al [107] combined topic identification, network analysis, and sentiment analysis to study misinformation-related Twitter discourse and user behavior. Velásquez et al [108] used Latent Dirichlet Allocation (LDA)-based topic modeling across multiple platforms to examine how online hate communities incorporated COVID-19 into malicious narratives and showed how platform moderation policies could influence the spread of such content. These studies demonstrate the value of topic modeling and thematic analysis for mapping discursive structure, tracing narrative evolution, and linking misinformation to broader community dynamics.

#### Sentiment and Emotion Analysis

A second major line of work focuses on affective expression in social media discourse. Sentiment and emotion analysis are used to examine how fear, distrust, support, skepticism, and related emotional responses emerge in discussions surrounding vaccines, health policies, and misinformation. Prabagar et al [95] applied a multimodule AI framework to analyze emotional shifts in approximately 400,000 vaccine-related posts from Twitter and Reddit. Thakur [109] used the Valence Aware Dictionary and Sentiment Reasoner (VADER) to classify positive and negative sentiment in a large Twitter dataset, while Satu et al [83] used an ML classification model and other models to identify the most frequent topics and their associated sentiment categories across 6 datasets. These studies show that misinformation is not only informational in nature but also affective, and that sentiment-based methods can help capture the emotional conditions under which misinformation gains visibility and influence.

#### False Information Detection and Classification

A substantial portion of the reviewed literature is devoted to the automated detection and classification of false information, including misinformation, disinformation, rumors, and fake news. Although the terminology varies across studies, the underlying methodological objective is similar: to distinguish misleading from reliable content at scale using textual, structural, network-based, or hybrid features. Ajao et al [110] tested multiple ML and DL models for detecting pandemic-related misinformation and found that support vector machine (SVM) outperformed RNN in their setting. Fedoruk et al [111] proposed the Plebeian algorithm, which combines sentiment analysis and postpopularity with a democratic moderation mechanism, thereby linking detection to platform governance. Gupta and Potika [112] examined the dissemination of fake versus real news through social network analysis and community detection, while Qureshi et al [113] found that hybrid features outperformed individual network or user features in source-based fake news detection. Other studies focus more directly on performance optimization. A total of 3 studies [114-116] compared a wide range of ML, DL, and transformer-based approaches to improve classification accuracy. Iwendi et al [117] demonstrated the value of richer feature spaces and domain-specific embeddings, while Nassif et al [118] showed the importance of language-specific contextual embedding models in Arabic fake news detection. Taken together, these studies reflect a strong technical orientation toward automating authenticity assessment. At the same time, they also suggest that classification performance alone is insufficient to address the broader sociotechnical challenges of misinformation, particularly when interpretability, governance, and real-world deployment are taken into account.

#### Multilingual and Cross-Lingual Analysis

The multilingual nature of social media substantially increases the complexity of COVID-19 misinformation research. Because misleading narratives circulate across linguistic communities, approaches developed for English alone are insufficient for capturing the global scope of the infodemic. The reviewed literature includes work in Arabic [86,118,119], Chinese [51,52], Japanese [73], Sinhala [103], Persian [120], and other languages, highlighting the need for methods that are robust beyond English-centric settings. For example, Caldera et al [103] used Sinhala tweets to identify hate-speech trends and applied time-series analysis to predict commenting behavior. Ashraf et al [121] proposed an ML-based rumor detection model for English and Arabic COVID-19 tweets using TF-IDF features and multiple classifiers. In a related study, Almars et al [122] introduced a hybrid attention neural network combining CNN and Bi-LSTM to detect and interpret rumors in Arabic tweets. These studies show that multilingual and cross-lingual analysis requires more than simple language transfer; it often demands tailored feature design, annotation strategies, and model architectures. More broadly, this strand of the literature underscores that methodological robustness in misinformation research depends on sensitivity to linguistic and cultural variation. The English-language eligibility criterion applied to article publication, not to dataset language. The languages named here are illustrative examples from the subsection, not a complete census of the primary dataset languages across all 100 studies; the retained records do not support a reliable study-level language distribution.

Overall, the reviewed literature demonstrates the value of computational methods for processing large-scale online misinformation data. However, it also reveals a detection-centered orientation. Most studies use computational models to identify, classify, cluster, or describe misinformation-related content, while fewer studies connect these outputs to downstream governance tasks such as moderation, warning design, fact-checking workflows, public communication strategies, or intervention evaluation. This creates a gap between information processing and information governance. For online information research, the key challenge is not only whether misinformation can be detected accurately but also whether computational outputs can support explainable, actionable, and context-sensitive responses in real-world information environments. A related limitation is the insufficiently visible role of human-in-the-loop interpretation. Still, several included studies demonstrate ways to integrate human judgment with computational analysis. Dhungana Sainju et al [45] combined supervised ML with independent human coding and qualitative content analysis; Roe et al [74] paired automated sentiment analysis with questionnaire data; and Hagen et al [77] integrated network analysis and ML with manual inductive coding of community discourse. Other studies manually labeled Arabic tweets using categories derived from the Arabic Infectious Disease Ontology [86], evaluated explainable model outputs in a human-participant experiment [116], or developed detailed annotation guidelines for fine-grained, multilabel misinformation coding and assessed interannotator reliability using a second annotator on a 1500-tweet subsample [119]. These examples show that human expertise can contribute during annotation, validation, interpretation, and triangulation rather than only at the downstream governance stage. However, because human involvement was not retained as a separate study-level extraction variable, the review does not estimate its prevalence across the full corpus.

## Discussion

### Principal Findings

This review shows that COVID-19 misinformation research on social media has produced a substantial body of empirical knowledge, but that this knowledge remains unevenly developed from the perspective of infodemiology and online health information research. The literature has made important progress in detecting misinformation, classifying misleading content, identifying themes, and measuring sentiment. However, it remains less developed in explaining how misinformation circulates across platforms, gains credibility among users, interacts with platform affordances, and can be addressed through public health communication and governance-oriented interventions. These findings suggest that the field needs to move from a detection-centered orientation toward a broader infodemic governance agenda [123-125].

### Conceptual Fragmentation Limits Cumulative Knowledge

A first implication concerns conceptual clarity. The reviewed studies use terms such as misinformation, disinformation, fake news, rumor, and infodemic in overlapping ways. Although these terms are related, they differ in intent, accuracy, source, circulation pattern, and potential harm. When studies define and operationalize these concepts differently, it becomes difficult to compare findings, combine evidence, or evaluate the effectiveness of interventions across contexts. For example, a model trained to detect “fake news” may not capture rumor, satire, misleading framing, or sincere but inaccurate health beliefs. Similarly, a study of vaccine hesitancy may capture attitudes that are not necessarily misinformation. Future infodemiology research should adopt clearer conceptual distinctions and make explicit how definitions are translated into data collection, annotation, modeling, and evaluation [7,123]. Accordingly, this review preserves study-specific labels when describing individual papers while using misinformation as an explicitly defined umbrella term in the synthesis; source labels are not assumed to be conceptually equivalent.

### Platform Concentration Narrows Understanding of Online Information Disorder

A second implication concerns platform concentration. The reviewed literature is heavily weighted toward text-based and relatively accessible platforms, especially X/Twitter [39,47,77]. This concentration has practical advantages because such platforms provide large-scale public data suitable for computational analysis. However, it also narrows the field’s understanding of online misinformation. Misinformation also circulates through Facebook groups, Reddit communities, YouTube videos, Instagram posts, TikTok videos, messaging platforms, podcasts, and cross-platform information flows [69-71]. These environments differ in visibility, moderation, affordances, audience structure, and data accessibility. As a result, findings from X/Twitter-based studies should not be assumed to generalize to all online information environments. Future research should develop more comparative and cross-platform designs that examine how misinformation changes as it moves across platforms, media formats, and communities.

### Detection-Centered Computation Is Necessary but Insufficient

A third implication concerns the dominant role of detection-centered computation. NLP, ML, DL, topic modeling, and sentiment analysis have made it possible to study online misinformation at scale. These methods are valuable because they can identify misleading content, detect emerging narratives, measure affective patterns, and support large-scale monitoring. However, detection alone does not explain why misinformation becomes credible, how it spreads through social networks, how users interpret it, or which interventions reduce its influence. High classification accuracy also does not necessarily translate into real-world usefulness if model outputs are not interpretable, timely, culturally sensitive, or integrated into governance workflows. Future research should evaluate computational methods not only by predictive performance but also by their actionability for public communication, content moderation, fact-checking, and institutional response [126-128].

### Comparison With Prior Work

Prior reviews provide important foundations for understanding COVID-19 misinformation on social media. Gabarron et al [129] conducted a systematic review of COVID-19-related misinformation on social media, Joseph et al [130] produced a scoping review of COVID-19 misinformation on social media, Sanaullah et al [131] systematically reviewed ML applications for COVID-19 misinformation, and Adebesin et al [132] used bibliometric analysis to examine social media’s role in health misinformation and disinformation during the pandemic. The present study does not claim to be the first or an exhaustive review. Its distinct contribution is to integrate 4 dimensions within a focused corpus of 100 empirical studies from the peak-pandemic period: conceptual definitions and operationalizations; topic, platform, population, and language coverage; the end-to-end computational pipeline from data acquisition through modeling; and the gap between detection-oriented outputs and actionable infodemic governance. This joint synthesis connects public health infodemiology with computational social media research and clarifies how review scope and methodological choices shape the gaps that become visible.

### Implications for Infodemic Governance

Table 1 summarizes the proposed shift from detection-centered misinformation research toward infodemic governance. The findings point toward an infodemic governance agenda for online misinformation research. Such an agenda treats misinformation not only as false content to be detected but also as a sociotechnical phenomenon shaped by information quality, platform design, algorithmic amplification, user interpretation, institutional credibility, and intervention capacity [123,125]. This perspective highlights several priorities. First, research should connect misinformation detection to intervention design, including warning labels, correction strategies, fact-checking systems, moderation policies, and public communication campaigns [126,127]. Second, studies should examine diffusion and source tracing to understand where misinformation originates, how it gains visibility, and how it moves across platforms. Third, future work should address multilingual, multimodal, and community-specific misinformation, especially in underrepresented languages and media formats. Fourth, research should develop evaluation criteria that go beyond accuracy to include explainability, timeliness, fairness, robustness, and usefulness for decision-making.

**Table 1.** From detection-centered misinformation research to infodemic governance.

Current limitation	Needed shift	Research opportunities
Inconsistent definitions	Conceptual standardization	Distinguish misinformation, disinformation, rumor, fake news, and infodemic-related content; report how concepts are operationalized in data collection, labeling, and modeling.
Platform concentration	Cross-platform analysis	Compare misinformation across X, formerly known as Twitter, Facebook, Reddit, YouTube, TikTok, Instagram, and messaging platforms; examine cross-platform narrative migration.
Text-centered data	Multimodal information analysis	Study images, videos, memes, audio, hyperlinks, and mixed media misinformation in addition to textual posts.
Detection-centered models	Governance-oriented systems	Link detection outputs to moderation, fact-checking, warning labels, public communication, and intervention workflows.
Static snapshots	Longitudinal and diffusion analysis	Track how misinformation emerges, spreads, mutates, and declines over time; examine source tracing and amplification pathways.
Accuracy-focused evaluation	Actionability-focused evaluation	Evaluate models by explainability, timeliness, fairness, robustness, cultural sensitivity, and usefulness for decision-making.

### Limitations

This review has several limitations. First, the review scope was restricted to English-language publications published between February 2020 and October 2022. Studies published after October 2022 were excluded. The publication-language restriction did not exclude studies solely because their underlying social media datasets were non-English. However, dataset language was not retained as a complete study-level variable, so the review cannot quantify the full distribution of languages represented in the corpus. The selected time window captures the peak pandemic period and allows us to examine how online misinformation research developed during an intensive phase of public health uncertainty and platform-mediated communication. However, it does not capture more recent developments in misinformation research, including postpandemic platform changes and the rise of generative AI-enabled misinformation. Second, the literature search was limited to PubMed and IEEE Xplore. These databases were selected to capture the intersection of public health misinformation research and computational social media analysis. However, relevant studies indexed in information science, communication, management, and social science databases may not have been included. For this reason, the review should be interpreted as a structured synthesis of a focused multidisciplinary corpus, not a comprehensive systematic review of all COVID-19 misinformation research. A related methodological limitation concerns the completeness of the retained search and study-selection documentation. Although Figure 1 reports author-verified aggregate identification, preliminary-screening, eligibility, duplicate-removal, and final-inclusion counts, together with the principal eligibility exclusion categories, a complete contemporaneous item-level screening log and record-level exclusion reasons for every record not retained during preliminary screening were not preserved. The complete original database-specific query syntax was also not preserved. Consequently, exact replication of the searches and independent auditing of all study-selection decisions at the individual-record level are limited. Figure 1 should be interpreted as an aggregate study-selection summary, not as a complete PRISMA flow diagram.

Third, the targeted search used the exact topic terms “misinformation,” “disinformation,” “fake news,” “rumor,” and “infodemic.” Adjacent concepts such as inaccurate information, unreliable information, low-quality information, conspiracy theories, propaganda, extremism, information disorder, or platform manipulation were not searched unless an included title or abstract also used one of the specified terms. Relevant studies using only adjacent terminology may have been omitted. Fourth, the review intentionally privileged computational, quantitative, and social media analytic studies and excluded noncomputational behavioral research, as well as studies focused solely on nontextual image or video content. The corpus’s detection-centered and text-centered profile partly reflects the eligibility criteria and should not be interpreted as a prevalence estimate for the entire COVID-19 misinformation field. Fifth, the corpus is weighted toward conversational, text-based platforms, particularly X/Twitter, Reddit, Weibo, and Facebook, whereas image-centric, video-based, audio-based, and closed messaging environments are less represented. Differential platform access and data openness likely contributed to this imbalance, further limiting generalization across online information environments.

### Conclusions

This peak-pandemic scoping review synthesized 100 empirical studies on COVID-19 misinformation on social media published between February 2020 and October 2022. We examined this literature as an infodemiology and online health information research domain concerned with how misinformation was conceptualized, measured, analyzed, and governed in social media environments during an intensive phase of public health uncertainty and platform-mediated communication.

The review identified 3 main patterns. First, the literature applies inconsistent definitions and operationalizations of misinformation, disinformation, fake news, rumor, and infodemic-related content. This conceptual fragmentation limits comparability across studies and complicates cumulative knowledge development. Second, the literature is concentrated around a limited set of topics and platforms, especially vaccine-related discourse and X/Twitter. Although this concentration reflects the urgency, platform data availability, and eligibility criteria of the review period, it narrows understanding of how misinformation operates across different platforms, communities, languages, and media modalities. Third, computational research has focused predominantly on detection, classification, topic modeling, and sentiment analysis. These approaches are valuable, but they provide only a partial basis for understanding and governing online misinformation.

The central contribution of this review is to reframe COVID-19 misinformation research as a sociotechnical infodemic governance problem. Online misinformation is not only false or misleading content to be detected; it is also shaped by platform affordances, algorithmic amplification, user interpretation, institutional trust, information quality, and intervention capacity. Future research should move beyond detection-centered models to examine diffusion, source tracing, cross-platform movement, multilingual and multimodal misinformation, and the effectiveness of governance-oriented interventions. By mapping the conceptual, contextual, and computational patterns of the literature, this study provides a foundation for more robust, inclusive, and actionable research on misinformation in online health information environments.

## Supplementary material

10.2196/79434 Multimedia Appendix 1 Search strategy and PRISMA-ScR (Preferred Reporting Items for Systematic Reviews and Meta-Analyses extension for Scoping Reviews) informed reporting notes.

10.2196/79434 Checklist 1 PRISMA-ScR checklist.

## Abbreviations

5G: fifth-generation
BERT: Bidirectional Encoder Representations from Transformers
Bi-GRUs: bidirectional gated recurrent units
Bi-LSTM: bidirectional long short-term memory
BoW: bag of words
CCFD: community-contributed COVID-19 fact data
CNN: convolutional neural network
DL: deep learning
GRU: gated recurrent units
KNN: k-nearest neighbors
LDA: Latent Dirichlet Allocation
LSTM: long short-term memory
ML: machine learning
NB: naïve Bayesian
NLP: natural language processing
PCA: principal component analysis
PRISMA: Preferred Reporting Items for Systematic Reviews and Meta-Analyses
PRISMA-ScR: Preferred Reporting Items for Systematic Reviews and Meta-Analyses extension for Scoping Reviews
RNN: recurrent neural network
RQ: research question
SVM: support vector machine
TF-IDF: term frequency-inverse document frequency
VADER: Valence Aware Dictionary and Sentiment Reasoner
XGBoost: extreme gradient boosting

## References

[1] Boyd D (2010). Social Network Sites as Networked Publics: Affordances, Dynamics, and Implications.

[2] Treem JW, Leonardi PM (2013). Social media use in organizations: exploring the affordances of visibility, editability, persistence, and association. Ann Int Commun Assoc.

[3] Gillespie T, Boczkowski PJ, Foot KA (2014). Media Technologies: Essays on Communication, Materiality, and Society.

[4] Kulshrestha J, Eslami M, Messias J Quantifying search bias: investigating sources of bias for political searches in social media.

[5] Cinelli M, De Francisci Morales G, Galeazzi A, Quattrociocchi W, Starnini M (2021). The echo chamber effect on social media. Proc Natl Acad Sci U S A.

[6] Törnberg P (2018). Echo chambers and viral misinformation: modeling fake news as complex contagion. PLoS One.

[7] Wardle C (2020). Understanding information disorder: essential guides. https://firstdraftnews.org/wp-content/uploads/2019/10/Information_Disorder_Digital_AW.pdf.

[8] Kim A, Moravec PL, Dennis AR (2019). Combating fake news on social media with source ratings: the effects of user and expert reputation ratings. J Manag Inf Syst.

[9] Garrett L (2020). COVID-19: the medium is the message. Lancet.

[10] Chon MG, Kim S (2022). Dealing with the COVID-19 crisis: theoretical application of social media analytics in government crisis management. Public Relat Rev.

[11] Chen Q, Min C, Zhang W, Wang G, Ma X, Evans R (2020). Unpacking the black box: how to promote citizen engagement through government social media during the COVID-19 crisis. Comput Human Behav.

[12] Tsao SF, Chen H, Tisseverasinghe T, Yang Y, Li L, Butt ZA (2021). What social media told us in the time of COVID-19: a scoping review. Lancet Digit Health.

[13] Cinelli M, Quattrociocchi W, Galeazzi A (2020). The COVID-19 social media infodemic. Sci Rep.

[14] Borges do Nascimento IJ, Pizarro AB, Almeida JM (2022). Infodemics and health misinformation: a systematic review of reviews. Bull World Health Organ.

[15] Liu Y, Whitfield C, Zhang T, Hauser A, Reynolds T, Anwar M (2021). Monitoring COVID-19 pandemic through the lens of social media using natural language processing and machine learning. Health Inf Sci Syst.

[16] Joy SKS, Dofadar DF, Khan RH, Ahmed MS, Rahman R A comparative study on covid-19 fake news detection using different transformer based models.

[17] Khan S, Hakak S, Deepa N, Prabadevi B, Dev K, Trelova S (2021). Detecting COVID-19-related fake news using feature extraction. Front Public Health.

[18] Priya A, Kumar A (2021). Deep ensemble approach for COVID-19 fake news detection from social media.

[19] F IMA, Yaddarabullah Y, Permana SDH Classification hoax news of COVID-19 on instagram using k-nearest neighbor.

[20] Li X, Lu P, Hu L, Wang X, Lu L (2022). A novel self-learning semi-supervised deep learning network to detect fake news on social media. Multimed Tools Appl.

[21] Zarocostas J (2020). How to fight an infodemic. The Lancet.

[22] (2013). Global risks 2013: eighth edition. https://www3.weforum.org/docs/WEF_GlobalRisks_Report_2013.pdf.

[23] Wang Y, McKee M, Torbica A, Stuckler D (2019). Systematic literature review on the spread of health-related misinformation on social media. Soc Sci Med.

[24] Tasnim S, Hossain MM, Mazumder H (2020). Impact of rumors and misinformation on COVID-19 in social media. J Prev Med Public Health.

[25] Valente A, Holanda M, Mariano AM, Furuta R, Da Silva D Analysis of academic databases for literature review in the computer science education field.

[26] Stevens H, Rasul ME, Oh YJ (2022). Emotions and incivility in vaccine mandate discourse: natural language processing insights. JMIR Infodemiology.

[27] Lanyi K, Green R, Craig D, Marshall C (2021). COVID-19 vaccine hesitancy: analysing Twitter to identify barriers to vaccination in a low uptake region of the UK. Front Digit Health.

[28] Fallis D (2015). What is disinformation?. Libr Trends.

[29] Tandoc EC, Lim ZW, Ling R (2018). Defining “fake news” a typology of scholarly definitions. Digital J.

[30] Allcott H, Gentzkow M (2017). Social media and fake news in the 2016 election. J Econ Perspect.

[31] Rastogi S, Bansal D (2022). Disinformation detection on social media: an integrated approach. Multimed Tools Appl.

[32] Bahja M, Safdar GA (2020). Unlink the link between COVID-19 and 5G networks: an NLP and SNA based approach. IEEE Access.

[33] Bangyal WH, Qasim R, Rehman NU (2021). Detection of fake news text classification on COVID-19 using deep learning approaches. Comput Math Methods Med.

[34] Al-Ramahi M, Elnoshokaty A, El-Gayar O, Nasralah T, Wahbeh A (2021). Public discourse against masks in the COVID-19 era: infodemiology study of Twitter data. JMIR Public Health Surveill.

[35] Jang H, Rempel E, Roth D, Carenini G, Janjua NZ (2021). Tracking COVID-19 discourse on Twitter in North America: infodemiology study using topic modeling and aspect-based sentiment analysis. J Med Internet Res.

[36] Park S, Han S, Kim J (2021). COVID-19 discourse on Twitter in four Asian countries: case study of risk communication. J Med Internet Res.

[37] de Oliveira DVB, Albuquerque UP (2021). Cultural evolution and digital media: diffusion of fake news about COVID-19 on Twitter. SN Comput Sci.

[38] Weinzierl MA, Harabagiu SM (2021). Automatic detection of COVID-19 vaccine misinformation with graph link prediction. J Biomed Inform.

[39] Ke SY, Neeley-Tass ES, Barnes M, Hanson CL, Giraud-Carrier C, Snell Q (2022). COVID-19 health beliefs regarding mask wearing and vaccinations on Twitter: deep learning approach. JMIR Infodemiology.

[40] Hayawi K, Shahriar S, Serhani MA, Taleb I, Mathew SS (2022). ANTi-Vax: a novel Twitter dataset for COVID-19 vaccine misinformation detection. Public Health.

[41] Shang L, Kou Z, Zhang Y, Chen J, Wang D A privacy-aware distributed knowledge graph approach to qois-driven COVID-19 misinformation detection.

[42] Guntuku SC, Buttenheim AM, Sherman G, Merchant RM (2021). Twitter discourse reveals geographical and temporal variation in concerns about COVID-19 vaccines in the United States. Vaccine.

[43] Sauvayre R, Vernier J, Chauvière C (2022). An analysis of French-language Tweets about COVID-19 vaccines: supervised learning approach. JMIR Med Inform.

[44] Bari A, Heymann M, Cohen RJ (2022). Exploring Coronavirus disease 2019 vaccine hesitancy on Twitter using sentiment analysis and natural language processing algorithms. Clin Infect Dis.

[45] Dhungana Sainju K, Zaidi H, Mishra N, Kuffour A (2022). Xenophobic bullying and COVID-19: an exploration using big data and qualitative analysis. Int J Environ Res Public Health.

[46] Farrell T, Gorrell G, Bontcheva K (2020). Vindication, virtue, and vitriol: a study of online engagement and abuse toward British MPs during the COVID-19 pandemic. J Comput Soc Sci.

[47] Gerts D, Shelley CD, Parikh N (2021). “Thought I’d Share First” and other conspiracy theory Tweets from the COVID-19 infodemic: exploratory study. JMIR Public Health Surveill.

[48] Ng JY, Abdelkader W, Lokker C (2022). Tracking discussions of complementary, alternative, and integrative medicine in the context of the COVID-19 pandemic: a month-by-month sentiment analysis of Twitter data. BMC Complement Med Ther.

[49] Melton CA, White BM, Davis RL, Bednarczyk RA, Shaban-Nejad A (2022). Fine-tuned sentiment analysis of COVID-19 vaccine-related social media data: comparative study. J Med Internet Res.

[50] Han Y Identification and analysis of opinion leaders for COVID-19 related rumor spreading in social media. https://ieeexplore.ieee.org/abstract/document/9898926.

[51] Leng Y, Zhai Y, Sun S (2021). Misinformation during the COVID-19 outbreak in China: cultural, social and political entanglements. IEEE Trans Big Data.

[52] Lu HY, Fan C, Song X, Fang W (2021). A novel few-shot learning based multi-modality fusion model for COVID-19 rumor detection from online social media. PeerJ Comput Sci.

[53] Shi A, Qu Z, Jia Q, Lyu C Rumor detection of COVID-19 pandemic on online social networks.

[54] Liu Y (2020). A comparative study of vector space language models for sentiment analysis using reddit data. Master’s thesis.

[55] Melton CA, Olusanya OA, Ammar N, Shaban-Nejad A (2022). Sentiment analysis of the Covid-19 vaccines on social media. Stud Health Technol Inform.

[56] Patel A, Meehan K Fake news detection on reddit utilising countvectorizer and term frequency-inverse document frequency with logistic regression, multinominalnb and support vector machine.

[57] Alouffi B, Alharbi A, Sahal R, Saleh H (2021). An optimized hybrid deep learning model to detect COVID-19 misleading information. Comput Intell Neurosci.

[58] Catapang JK, Cleofas JV Topic modeling, clade-assisted sentiment analysis, and vaccine brand reputation analysis of COVID-19 vaccine-related Facebook comments in the Philippines.

[59] Maakoul O, Boucht S, El Hachimi K, Azzouzi S Towards evaluating the COVID’19 related fake news problem: case of morocco. https://ieeexplore.ieee.org/xpl/mostRecentIssue.jsp?punumber=9314356.

[60] Sear RF, Velasquez N, Leahy R (2020). Quantifying COVID-19 content in the online health opinion war using machine learning. IEEE Access.

[61] Kirn SL, Hinders MK (2022). Ridge count thresholding to uncover coordinated networks during onset of the Covid-19 pandemic. Soc Netw Anal Min.

[62] Abdelminaam DS, Ismail FH, Taha M, Taha A, Houssein EH, Nabil A (2021). CoAID-DEEP: an optimized intelligent framework for automated detecting COVID-19 misleading information on Twitter. IEEE Access.

[63] Abe T, Higashi D, Nishiyama D, Toriumi F, Galam S, Ishii A The toilet paper issue during the COVID-19 crisis.

[64] Al-Rakhami MS, Al-Amri AM (2020). Lies kill, facts save: detecting COVID-19 misinformation in Twitter. IEEE Access.

[65] Trezza D (2023). To scrape or not to scrape, this is dilemma. The post-API scenario and implications on digital research. Front Sociol.

[66] Shahsavari S, Holur P, Wang T, Tangherlini TR, Roychowdhury V (2020). Conspiracy in the time of corona: automatic detection of emerging COVID-19 conspiracy theories in social media and the news. J Comput Soc Sci.

[67] Bhattacharyya S Classification and analysis of general misinformation and COVID-related misinformation within subreddits of opposing political viewpoints.

[68] Xue H, Gong X, Stevens H (2022). COVID-19 vaccine fact-checking posts on Facebook: observational study. J Med Internet Res.

[69] Zarei K, Farahbakhsh R, Crespi N, Tyson G (2021). Dataset of coronavirus content from Instagram with an exploratory analysis. IEEE Access.

[70] Wang AW, Lan JY, Wang MH, Yu C (2021). The evolution of rumors on a closed social networking platform during COVID-19: algorithm development and content study. JMIR Med Inform.

[71] Ginossar T, Cruickshank IJ, Zheleva E, Sulskis J, Berger-Wolf T (2022). Cross-platform spread: vaccine-related content, sources, and conspiracy theories in YouTube videos shared in early Twitter COVID-19 conversations. Hum Vaccin Immunother.

[72] Kwok SWH, Vadde SK, Wang G (2021). Tweet topics and sentiments relating to COVID-19 vaccination among Australian Twitter users: machine learning analysis. J Med Internet Res.

[73] Niu Q, Liu J, Kato M (2022). Public opinion and sentiment before and at the beginning of COVID-19 vaccinations in Japan: Twitter analysis. JMIR Infodemiology.

[74] Roe C, Lowe M, Williams B, Miller C (2021). Public perception of SARS-CoV-2 vaccinations on social media: questionnaire and sentiment analysis. Int J Environ Res Public Health.

[75] Wahbeh A, Nasralah T, Al-Ramahi M, El-Gayar O (2020). Mining physicians’ opinions on social media to obtain insights into COVID-19: mixed methods analysis. JMIR Public Health Surveill.

[76] Benis A, Chatsubi A, Levner E, Ashkenazi S (2021). Change in threads on Twitter regarding influenza, vaccines, and vaccination during the COVID-19 pandemic: artificial intelligence-based infodemiology study. JMIR Infodemiology.

[77] Hagen L, Fox A, O’Leary H (2022). The role of influential actors in fostering the polarized COVID-19 vaccine discourse on Twitter: mixed methods of machine learning and inductive coding. JMIR Infodemiology.

[78] Liu Y, Anwar M Learning programming in social media: an nlp-powered reddit study.

[79] Sharma U, Pandey P, Kumar S (2022). A transformer-based model for evaluation of information relevance in online social-media: a case study of Covid-19 media posts. New Gener Comput.

[80] Clamor TDS, Solano GA, Oco N, Catapang JK, Cleofas J, Isip-Tan IT Identification and analysis of COVID-19-related misinformation tweets via kullback-leibler divergence for informativeness and phraseness and biterm topic modeling.

[81] Liu Y, Yue Z, Anwar M Monkeypox at-a-glance from google trends and reddit. https://ieeexplore.ieee.org/abstract/document/9983632.

[82] Whitfield C, Liu Y, Anwar M Surveillance of COVID-19 pandemic using social media: a reddit study in north carolina.

[83] Satu MS, Khan MI, Mahmud M (2021). TClustVID: a novel machine learning classification model to investigate topics and sentiment in COVID-19 tweets. Knowl Based Syst.

[84] Sharma MK, Kumar P, Rasool A, Dubey A, Mahto VK Classification of actual and fake news in pandemic.

[85] Shushkevich E, Cardiff J Detecting fake news about covid-19 on small datasets with machine learning algorithms.

[86] Alsudias L, Rayson P (2021). Social media monitoring of the COVID-19 pandemic and influenza epidemic with adaptation for informal language in Arabic Twitter data: qualitative study. JMIR Med Inform.

[87] Liu Z, Lin Y, Sun M (2020). Representation Learning for Natural Language Processing.

[88] Chowdhury AN, Guha S, Amin N, Khan SI Exploiting ensemble of transformer models for detecting informative tweets.

[89] Mourad A, Srour A, Harmanani H, Jenainati C, Arafeh M (2020). Critical impact of social networks infodemic on defeating Coronavirus COVID-19 pandemic: Twitter-based study and research directions. IEEE Trans Netw Serv Manage.

[90] Michel K, Smith M, Brown B, King M, Dozier G A study of social network messages during the COVID-19 infodemic: salient features and the propagation of information types.

[91] Narra M, Umer M, Sadiq S (2022). Selective feature sets based fake news detection for COVID-19 to manage infodemic. IEEE Access.

[92] Mahbub S, Pardede E, Kayes ASM (2022). COVID-19 rumor detection using psycho-linguistic features. IEEE Access.

[93] Hua Y, Jiang H, Lin S (2022). Using Twitter data to understand public perceptions of approved versus off-label use for COVID-19-related medications. J Am Med Inform Assoc.

[94] Micallef N, He B, Kumar S, Ahamad M, Memon N The role of the crowd in countering misinformation: a case study of the COVID-19 infodemic.

[95] Prabagar K, Srikandabala K, Loganathan N Investigating COVID-19 vaccine messaging in online social networks using artificial intelligence.

[96] Ramakrishnan K, Balakrishnan V SentiMage: a sentiment-image-based covid-19 health misinformation detection using machine learning.

[97] Smith GY, Kabban CMS, Hopkinson KM, Oxley ME, Noel GE, Cheng H Sensor fusion for context analysis in social media covid-19 data.

[98] Elhadad MK, Li KF, Gebali F (2020). Detecting misleading information on COVID-19. IEEE Access.

[99] Röchert D, Shahi GK, Neubaum G, Ross B, Stieglitz S (2021). The networked context of COVID-19 misinformation: informational homogeneity on YouTube at the beginning of the pandemic. Online Soc Netw Media.

[100] Varshney D, Vishwakarma DK Analysing and identifying crucial evidences for the prediction of false information proliferated during covid-19 outbreak: a case study.

[101] Hande A, Puranik K, Priyadharshini R, Thavareesan S, Chakravarthi BR Evaluating pretrained transformer-based models for covid-19 fake news detection.

[102] Sari AM, Ariyani NF, Ahmadiyah AS Evaluating the preliminary models to identify fake news on COVID-19 tweets.

[103] Caldera HMM, Meedin GSN, Perera I Time series based trend analysis for hate speech in twitter during COVID 19 pandemic.

[104] Kar D, Bhardwaj M, Samanta S, Azad AP No rumours please! a multi-indic-lingual approach for covid fake-tweet detection.

[105] Saghayan MH, Ebrahimi SF, Bahrani M Exploring the impact of machine translation on fake news detection: a case study on persian tweets about covid-19.

[106] Vogel I, Meghana M Detecting fake news spreaders on twitter from a multilingual perspective.

[107] Kalantari N, Liao D, Motti VG Characterizing the online discourse in twitter: users’ reaction to misinformation around COVID-19 in twitter.

[108] Velásquez N, Leahy R, Restrepo NJ (2021). Online hate network spreads malicious COVID-19 content outside the control of individual social media platforms. Sci Rep.

[109] Thakur N (2022). MonkeyPox2022Tweets: a large-scale Twitter dataset on the 2022 Monkeypox outbreak, findings from analysis of Tweets, and open research questions. Infect Dis Rep.

[110] Ajao O, Garg A, Da Costa-Abreu M Exploring content-based and meta-data analysis for detecting fake news infodemic: a case study on COVID-19.

[111] Fedoruk B, Nelson H, Frost R, Fucile Ladouceur K (2021). The plebeian algorithm: a democratic approach to censorship and moderation. JMIR Form Res.

[112] Gupta K, Potika K Fake news analysis and graph classification on a covid-19 twitter dataset.

[113] Qureshi KA, Malick RAS, Sabih M, Cherifi H (2021). Complex network and source inspired COVID-19 fake news classification on Twitter. IEEE Access.

[114] Fatima R, Samad Shaikh N, Riaz A (2022). A natural language processing (NLP) evaluation on COVID-19 rumour dataset using deep learning techniques. Comput Intell Neurosci.

[115] Khan Y, Thakur S Fake news detection of south african COVID-19 related tweets using machine learning.

[116] Ayoub J, Yang XJ, Zhou F (2021). Combat COVID-19 infodemic using explainable natural language processing models. Inf Process Manag.

[117] Iwendi C, Mohan S, Khan S, Ibeke E, Ahmadian A, Ciano T (2022). Covid-19 fake news sentiment analysis. Comput Electr Eng.

[118] Nassif AB, Elnagar A, Elgendy O, Afadar Y (2022). Arabic fake news detection based on deep contextualized embedding models. Neural Comput Appl.

[119] Obeidat R, Gharaibeh M, Abdullah M, Alharahsheh Y (2022). Multi-label multi-class COVID-19 Arabic Twitter dataset with fine-grained misinformation and situational information annotations. PeerJ Comput Sci.

[120] Parvizimosaed M, Esnaashari M, Damia A, Bahmanyar R Using supervised learning models for creating a new fake news analysis and classification of a COVID-19 dataset: a case study on COVID-19 in iran.

[121] Ashraf N, Nayel H, Taha M A comparative study of machine learning approaches for rumors detection in covid-19 tweets.

[122] Almars AM, Almaliki M, Noor TH, Alwateer MM, Atlam E (2022). HANN: hybrid attention neural network for detecting Covid-19 related rumors. IEEE Access.

[123] Eysenbach G (2009). Infodemiology and infoveillance: framework for an emerging set of public health informatics methods to analyze search, communication and publication behavior on the Internet. J Med Internet Res.

[124] Mavragani A (2020). Infodemiology and infoveillance: scoping review. J Med Internet Res.

[125] Tangcharoensathien V, Calleja N, Nguyen T (2020). Framework for managing the COVID-19 infodemic: methods and results of an online, crowdsourced WHO technical consultation. J Med Internet Res.

[126] Loomba S, de Figueiredo A, Piatek SJ, de Graaf K, Larson HJ (2021). Measuring the impact of COVID-19 vaccine misinformation on vaccination intent in the UK and USA. Nat Hum Behav.

[127] Pennycook G, McPhetres J, Zhang Y, Lu JG, Rand DG (2020). Fighting COVID-19 misinformation on social media: experimental evidence for a scalable accuracy-nudge intervention. Psychol Sci.

[128] Pierri F, Perry BL, DeVerna MR (2022). Online misinformation is linked to early COVID-19 vaccination hesitancy and refusal. Sci Rep.

[129] Gabarron E, Oyeyemi SO, Wynn R (2021). COVID-19-related misinformation on social media: a systematic review. Bull World Health Organ.

[130] Joseph AM, Fernandez V, Kritzman S (2022). COVID-19 misinformation on social media: a scoping review. Cureus.

[131] Sanaullah AR, Das A, Das A, Kabir MA, Shu K (2022). Applications of machine learning for COVID-19 misinformation: a systematic review. Soc Netw Anal Min.

[132] Adebesin F, Smuts H, Mawela T, Maramba G, Hattingh M (2023). The role of social media in health misinformation and disinformation during the COVID-19 pandemic: bibliometric analysis. JMIR Infodemiology.

