# Supplementary material for: Online COVID-19 Misinformation and Infodemic Governance During the Peak Pandemic Period: Scoping Review of Social Media Research From 2020 to 2022
Source: JMIR Infodemiology. 2026 Oct 29;6:e79434. doi: 10.2196/79434 (PMC13718887; doi:10.2196/79434)
Supplement: Supplementary file 2 [file infodemiology-v6-e79434-s001.docx]

**Multimedia Appendix 1. Search Strategy and PRISMA-ScR-Informed Reporting Notes**

**Search Sources**

| **Database** | **Records retrieved** | **Coverage rationale** |
| --- | --- | --- |
| PubMed | 804 | Biomedical, public health, health communication, and infodemiology literature. |
| IEEE Xplore | 884 | Computer science, engineering, information systems, and computational social media literature. |
| Total | 1688 | Initial database search pool before duplicate checking and screening. |

**Core Search Logic**

- COVID-19 context: COVID-19, COVID19, COVID, coronavirus, or acute respiratory syndrome.
- Social media platform: social media, social network, Twitter, tweet, Reddit, Facebook, or Weibo.
- Misinformation topic: misinformation, disinformation, fake news, rumor, or infodemic.

Core Boolean string: ("COVID-19" OR "COVID19" OR "COVID" OR "coronavirus" OR "acute respiratory syndrome") AND ("social media" OR "social network" OR "Twitter" OR "tweet" OR "Reddit" OR "Facebook" OR "Weibo") AND ("misinformation" OR "disinformation" OR "fake news" OR "rumor" OR "infodemic").

**Supplementary Title/Abstract Search Audit**

To improve search transparency, we also report an author-provided Title/Abstract search audit. This audit is useful as supplementary documentation of the search logic and keyword sensitivity, but it should not be treated as a replacement for the main PRISMA identification count because it uses a narrower PubMed-style Title/Abstract field restriction and does not document duplicate removal or full-text exclusion reasons.

Audit query: ((Reddit[Title/Abstract]) OR (Twitter[Title/Abstract]) OR (Weibo[Title/Abstract]) OR (Social Media[Title/Abstract])) AND (COVID[Title/Abstract]) AND ((Rumor[Title/Abstract]) OR (fake news[Title/Abstract]) OR (misinformation[Title/Abstract]) OR (disinformation[Title/Abstract])).

**PRISMA-ScR Completion Notes**

The final included corpus contained 100 empirical studies selected from 1,688 database records. Aggregate intermediate counts are reported in Figure 1; however, a contemporaneous item-level screening log and record-level exclusion reasons were not preserved and are not reconstructed in this appendix.

| **Reporting area** | **Manuscript Location** | **Current Status** |
| --- | --- | --- |
| Title/abstract | Title and structured abstract | Revised to identify the manuscript as a PRISMA-informed scoping review and to use JMIR-style structured abstract headings. |
| Rationale/objectives | Introduction | Research gap and research questions stated explicitly. |
| Eligibility criteria | Methods | Inclusion and exclusion criteria stated |
| Information sources/search | Methods and Appendix 1 | Databases, dates, concepts, and core Boolean string stated. |
| Selection process | Methods and Figure 1 | Known counts reported; unlogged intermediate counts identified as not reconstructed. |
| Data charting/synthesis | Methods | Data extraction variables and thematic synthesis procedure described in the Methods. |
| Limitations | Discussion | Database, date, language, platform, keyword, and missing screening-log limitations stated. |
